# Deep Eutectic Solvent Pretreatment of Transgenic Biomass With Increased C_6_C_1_ Lignin Monomers

**DOI:** 10.3389/fpls.2019.01774

**Published:** 2020-01-29

**Authors:** Kwang Ho Kim, Yunxuan Wang, Masatsugu Takada, Aymerick Eudes, Chang Geun Yoo, Chang Soo Kim, Jack Saddler

**Affiliations:** ^1^ Clean Energy Research Center, Korea Institute of Science and Technology, Seoul, South Korea; ^2^ Department of Wood Science, University of British Columbia, Vancouver, BC, Canada; ^3^ Department of Paper and Bioprocess Engineering, State University of New York College of Environmental Science and Forestry, Syracuse, NY, United States; ^4^ Feedstocks Division, Joint BioEnergy Institute, Emeryville, CA, United States; ^5^ Environmental Genomics and Systems Biology Division, Lawrence Berkeley National Laboratory, Berkeley, CA, United States

**Keywords:** lignocellulosic biomass, lignin engineering, hydroxycinnamoyl-CoA hydratase-lyase, bioenergy, saccharification

## Abstract

The complex and heterogeneous polyphenolic structure of lignin confers recalcitrance to plant cell walls and challenges biomass processing for agroindustrial applications. Recently, significant efforts have been made to alter lignin composition to overcome its inherent intractability. In this work, to overcome technical difficulties related to biomass recalcitrance, we report an integrated strategy combining biomass genetic engineering with a pretreatment using a bio-derived deep eutectic solvent (DES). In particular, we employed biomass from an *Arabidopsis* line that expressed a bacterial hydroxycinnamoyl-CoA hydratase-lyase (HCHL) in lignifying tissues, which results in the accumulation of unusual C_6_C_1_ lignin monomers and a slight decrease in lignin molecular weight. The transgenic biomass was pretreated with renewable DES that can be synthesized from lignin-derived phenols. Biomass from the HCHL plant line containing C_6_C_1_ monomers showed increased pretreatment efficiency and released more fermentable sugars up to 34% compared to WT biomass. The enhanced biomass saccharification of the HCHL line is likely due to a reduction of lignin recalcitrance caused by the overproduction of C_6_C_1_ aromatics that act as degree of polymerization (DP) reducers and higher chemical reactivity of lignin structures with such C_6_C_1_ aromatics. Overall, our findings demonstrate that strategic plant genetic engineering, along with renewable DES pretreatment, could enable the development of sustainable biorefinery.

## Introduction

Growing concerns over global warming and our over-dependence on fossil resources have forced society to demand sustainable and green products (Isikgor and Becer, 2015). Lignocellulosic biomass presents a promising source of renewable carbon, holding enormous potential for the production of chemicals and fuels. In order to utilize biomass as a source of renewable energy and chemicals, there have been significant efforts to develop an efficient process for biomass conversion (Demirbaş, 2001). Among several technologies developed within the biorefinery concept, the production of second-generation biofuels (e.g., bioethanol) from lignocellulosic biomass is well established and close to commercialization. Despite the successful demonstration of biomass conversion to biofuels, the production of cellulosic biofuels still encounters several technical challenges for achieving a sustainable energy future (Dale et al., 2014; Maity, 2015; Kim and Kim, 2018).

Lignin, an essential component of biomass that provides mechanical strength for upright growth and acts as a physical barrier against pathogens (Boudet, 2007), is often blamed for conferring recalcitrance to biomass against processing and utilization. In the typical biological conversion process, effective removal of lignin is crucial to maximize the utilization of carbohydrates to produce fuels and building block chemicals (Ding et al., 2012; Ragauskas et al., 2014). However, considering its complex and heterogeneous structure, hydrophobic character, and other intractable properties, lignin is one of the most challenging biomaterials to handle (Himmel, 2009).

During the last decades, researchers have developed several routes to overcome lignin-associated recalcitrance. For example, the lignin-first approach was introduced to extract the reactive lignin at the early stage of biomass fractionation, providing opportunities for the use of both lignin and carbohydrates (Renders et al., 2017). In contrast to the conventional carbohydrates-oriented pretreatment technologies of biomass, this new biorefinery scheme offers potential valorization routes for both lignin fractions and residual carbohydrates. While the above strategy focuses on the development of processes to overcome the technical difficulties related to lignin, another effort to reduce the biomass recalcitrance through genetic engineering has shown its effectiveness (Chen and Dixon, 2007; Bhagia et al., 2016; Thomas et al., 2017).

**Figure 1:**
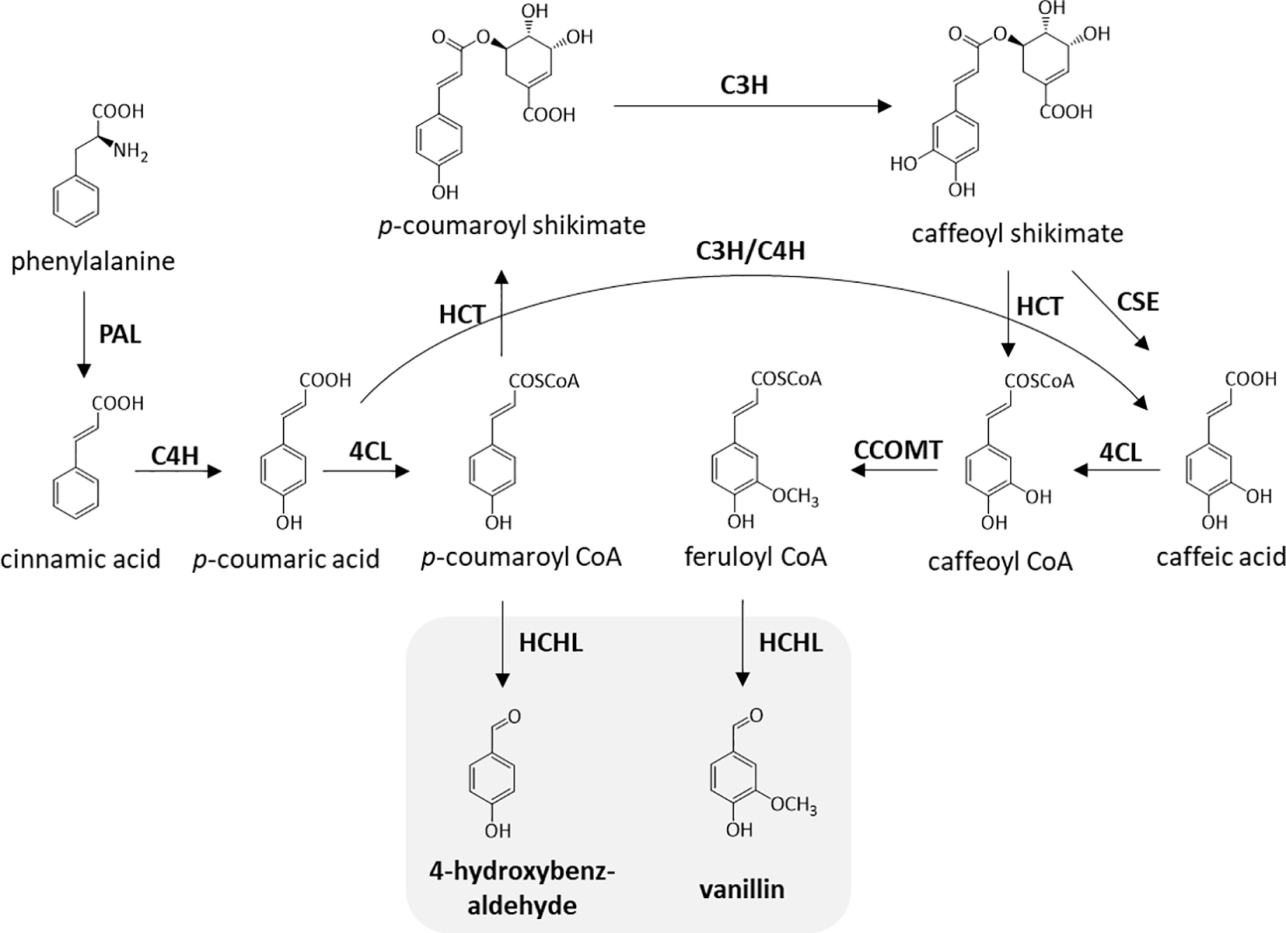
Enzymatic steps catalyzed by hydroxycinnamoyl-CoA hydratase-lyase (HCHL). The two products of HCHL activity (in gray) are converted into several other C6C1 aromatics in plant tissues. *PAL*, phenylalanine ammonia lyase; *C4H*, cinnamate 4-hydroxylase; *4CL*, 4-coumarate-CoA ligase; *HCT*, hydroxycinnamoyl-CoA shikimate hydroxycinnamoyl transferase; *C3H,* coumarate 3-hydroxylase; *CSE*, caffeoyl shikimate esterase; *CCOMT,* caffeoyl-CoA *O*-methyltransferase.

Previously, biomass cell wall engineering was directed to strategically reduce total lignin content by downregulating one or more enzymes in the monolignol pathway, which includes cinnamate 4-hydroxylase (*C4H*) (Schilmiller et al., 2009), 4-coumarate-CoA ligase (*4CL*) (Xu et al., 2011), and cinnamoyl-CoA reductase (*CCR*) (Chabannes et al., 2001). Although the decrease in the amount of lignin was proven to improve processing efficiency, this method often involves an agronomic penalty (Bonawitz and Chapple, 2013). Moreover, modification of lignin monomeric composition leading to structural modifications has been extensively studied to make biomass more amenable to processing without compromising biomass yield. In-planta expression of a bacterial 3-dehydroshikimate dehydratase resulted in the higher deposition of H-units and lower amounts of G- and S-units, resulting in more than a two-fold improvement in saccharification efficiency (Eudes et al., 2015). Incorporation of chemically labile ester linkages (zip-lignin) in the lignin backbone was proven to enhance biomass pretreatment efficiency (Wilkerson et al., 2014; Kim et al., 2017). Previous work also reported a strategy for the overproduction of uncommon lignin monomers through in-planta expression of a bacterial hydroxycinnamoyl-CoA hydratase-lyase (HCHL) in biomass (Mitra et al., 2002; Eudes et al., 2012). **Figure 1** describes the enzymatic reactions catalyzed by HCHL which is expressed in lignifying tissues of engineered plants. HCHL cleaves the side-chain of coumaroyl-CoA and feruloyl-CoA, resulting in an increased amount of unusual C_6_C_1_ end-groups in lignin, including 4-hydroxybenzaldehyde, vanillin, syringylaldehyde, and 4-hydroxybenzoic acid (Eudes et al., 2012).

Bio-derived deep eutectic solvents (DESs) have gained considerable attention because of their potential uses in biomass pretreatment and processing. DESs are mixtures of compounds formed by strong intermolecular hydrogen bonds, resulting in a lower melting point than that of any individual component (Dai et al., 2013). As an alternative to organic solvents for biomass pretreatment, DESs exhibit promising solvent properties including high dissolution capability, low vapor pressure, tunability, stabilization of carbohydrates through hydrogen-bond interactions, and compatibility with certain microorganisms (Vigier et al., 2015). Recently, DESs synthesized from lignin-derivable phenolic compounds were found to be effective in lignin removal, and thus resulted in enhanced biomass saccharification efficacy (Kim et al., 2018). Also, renewable DESs prepared from phenolic aldehydes (e.g., vanillin and 4-hydroxybenzaldehyde) with choline chloride (ChCl), integrated with the use of low-recalcitrant engineered biomass *via* down-regulation of cinnamyl alcohol dehydrogenase (CAD), demonstrated the potential of developing a closed-loop biorefinery process (Kim et al., 2019).

In this work, we employed, as a raw material for DES-assisted pretreatment, the biomass from a previously described plant genetic engineering approach that increases non-conventional C_6_C_1_ monomers in lignin *via* HCHL expression. We also demonstrate the use of renewable DES for pretreating such biomass designed for improved processability.

## Materials and Methods

### Biomass Material

*Arabidopsis thaliana* (ecotype Columbia, Col-0) wild type (WT) and line *IRX5:HCHL*-1 (HCHL) were used in this study (Eudes et al., 2012). Previously, five transgenic lines that express HCHL were isolated and characterized, of which two showed reduced biomass and/or height. Line *IRX5:HCHL*-1and one with no yield penalty was selected for in-depth characterization and grown on a larger scale for further research (Eudes et al., 2012). Seeds were germinated directly on soil. Growing conditions were: 14 h light/day at 100 µmol/m^2^/s, 22°C, and 55% humidity. For analyses, stems from mature senesced dried plants harvested without siliques and leaves were ball-milled to a fine powder using a Mixer Mill MM 400 (Retsch Inc., Newtown, PA) and stainless steel balls for 2 min.

### Bio-Derived Deep Eutectic Solvent Preparation

All chemicals used for DES synthesis were purchased from Sigma-Aldrich (St. Louis, MO) and used without further purification. DES used in this work was synthesized using ChCl (purity: ≥98%) and vanillin (VAN, purity: 99%) as a hydrogen-bond acceptor and donor, respectively. ChCl and VAN were mixed at 1:2 molar ratio, and then heated to 100°C with continuous stirring until a homogeneous liquid was formed (Kim et al., 2018; Kim et al., 2019). The prepared DES (i.e., ChCl-VAN) was stored in a vacuum oven before use.

### Pretreatment and Enzymatic Saccharification

For biomass pretreatment, 5 wt% biomass solution was prepared by mixing 0.2 g of biomass (WT and HCHL line) and 3.8 g ChCl-VAN in a 20 ml pressure tube (Ace Glass, Vineland, NJ). The tube was placed to the preheated oil bath at 80°C. A mild pretreatment temperature used in this work to examine the effect of engineered biomass on pretreatment efficiency (Kim et al., 2019). After 3 h, the pretreated slurry was transferred to a 15 ml centrifuge tube and washed with 50 ml (5 × 10 ml) ethanol and deionized water mixture (2:1, v/v) to separate lignin and remove any residual DES (Kim et al., 2019).

Enzymatic hydrolysis of the pretreated biomass was carried out in 5 ml of 50 mM citrate buffer supplemented with Cellic^®^ CTec3 (266.3 mg/ml) and HTec (234.8 mg/ml) from Novozymes (9:1 ratio) at 10 mg protein per gram solid biomass (solid loading ≈2 wt%). The enzymatic saccharification was performed in an Incubator-Genie™ rotating hybridization oven (Scientific Industries, Bohemia, NY) at 50°C for 72 h.

### Topochemical Analysis

Sections of intact stems of mature plants (5 cm from bottom) of unpretreated and DES-pretreated were gradually dehydrated with ethanol-water mixtures with increasing ethanol concentration up to 99.5%. After the ethanol was replaced by acetone, the samples were embedded in the epoxy resin. Ultrathin sections of 80 nm were prepared from embedded samples with a diamond knife mounted on ultramicrotome. Lignin was selectively stained with 1% KMnO_4_ and the stained section was mounted on copper grids, and observed with a Hitachi H7600 transmission electron microscope (TEM) at an acceleration voltage of 80 keV.

### Analytical Methods

Compositional analysis of raw materials and pretreated samples was conducted according to the National Renewable Energy Laboratory protocol (Sluiter et al., 2008), and the results are given in **Table 1**.

**Table 1:** Compositional analysis of wild-type (WT) and hydroxycinnamoyl-CoA hydratase-lyase (HCHL) *Arabidopsis* line.

	WT	HCHL
Glucan, wt%	28.9 (0.3)	28.7 (1.7)
Xylan, wt%	11.8 (0.0)	12.3 (0.6)
Lignin, wt%	21.9 (0.4)	19.6 (0.6)
Extractives, wt%	22.3 (0.3)	29.9 (2.1)*

The values in parentheses are standard deviations (statistical significance from WT *P < 0.05).

After pretreatment and saccharification, the hydrolysate was filtered through a 0.45 μm polytetrafluoroethylene (PTFE) syringe filter (VWR, Radnor, PA). The amount of glucose and xylose released was quantified using a YL 9100 high performance liquid chromatography (Young-Lin) equipped with a refractive index detector and a Bio-Rad Aminex HPX-87H ion-exchange column. A solution of 5 mM H_2_SO_4_ was used as a mobile phase at a constant flow rate of 0.6 ml/min, and the temperature of column compartment was maintained at 60°C.

Structural information of lignin was analyzed by two-dimensional (2D) ^1^H-^13^C heteronuclear single-quantum coherence (HSQC) nuclear magnetic resonance (NMR) spectroscopy. Before the NMR analysis, cellulolytic enzyme lignin was isolated from each line as described elsewhere (Yoo et al., 2016). The prepared lignin dissolved with DMSO-*d_6_* was placed in a 5 mm NMR tube. The NMR spectra were acquired using a Bruker Avance III HD 800 MHz NMR spectrometer equipped with a TCI Cryoprobe with the following acquisition parameters: spectra width 12 ppm in F2 (^1^H) dimension with 1024 time of domain (acquisition time, 53.2 ms), 200 ppm in F1 (^13^C) dimension with 512 time of domain (acquisition time, 6.4 ms), a 1.2-s relaxation delay, and 32 scans. Assignment of the HSQC spectra is described elsewhere (Kim and Ralph, 2010; Eudes et al., 2012).

Gel permeation chromatography (GPC) was used to determine the molecular weight distribution of the lignin from the WT control and HCHL transgenic. GPC measurement was conducted using a Waters 2489 GPC system equipped with a UV detector at 270 nm of wavelength. The eluent used for the analysis was tetrahydrofuran, and the three Waters Styragel columns (HR0.5, HR3, and HR4e) were used. All samples were acetylated using 1.0 ml acetic anhydride/pyridine (1:1, v/v) at room temperature for at least 24 h prior to GPC analysis.

### Computational Analysis

**Figure 2:**
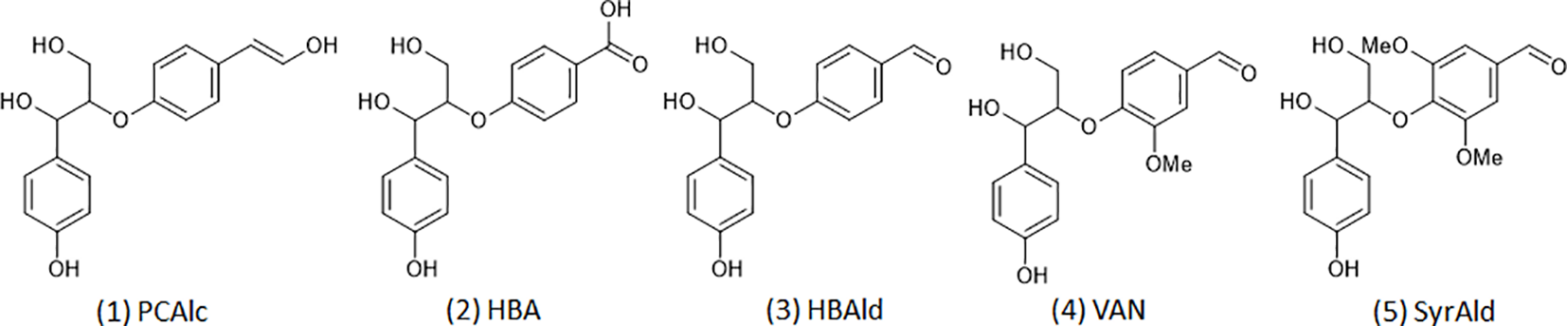
Dimeric model compounds: 1) 4-hydroxylphenylglycerol-β-*p*-coumaryl alcohol ether (PCAlc), 2) 4-hydroxylphenylglycerol-β-4-oxybenzoic acid ether (HBA), 3) 4-hydroxylphenylglycerol-β-4-oxybenzaldehyde ether (HBAld), 4) 4-hydroxylphenylglycerol-β-vanillin ether (VAN), and 5) 4-hydroxylphenylglycerol-β-syringaldehyde ether (SyrAld).

Density functional theory (DFT) based computational analysis was performed to gain a mechanistic understanding of structurally altered lignin molecules. It is noted, considering the complex structure of lignin, that lignin model compounds with a representative interunit linkage are typically employed for computational studies (Sun et al., 2016). **Figure 2** shows five representative β-O-4 dimeric model compounds from typical lignin (1) and from the C_6_C_1_-enriched lignin of the HCHL line (2, 3, 4, and 5) tested for the mechanistic study. The geometry optimizations of model compounds were conducted using DFT with the B3LYP and the 6-31+G(2d,2p) basis set. Frequency calculations were also carried out to verify that the optimized structures corresponded to energy minima. In this study, the electrophilicity index, a global reactivity descriptor, was calculated using the electronegativity and the chemical hardness to compare the chemical reactivity of each model structure (Shi et al., 2016; Kim et al., 2019).

### Statistical Analysis

Analysis of variance (ANOVA) was conducted using R (R Foundation for Statistical Computing, Vienna, Austria) to test the null hypothesis of no statistical difference in saccharification yield (glucose and xylose) between the WT and transgenic HCHL *Arabidopsis*. Three independent tests were conducted and each sample was analyzed. The null hypothesis was rejected at the 0.05 level.

## Results

### Structural Analysis of Lignin in Wild-Type and Hydroxycinnamoyl-Coa Hydratase-Lyase *Arabidopsis*

**Figure 3:**
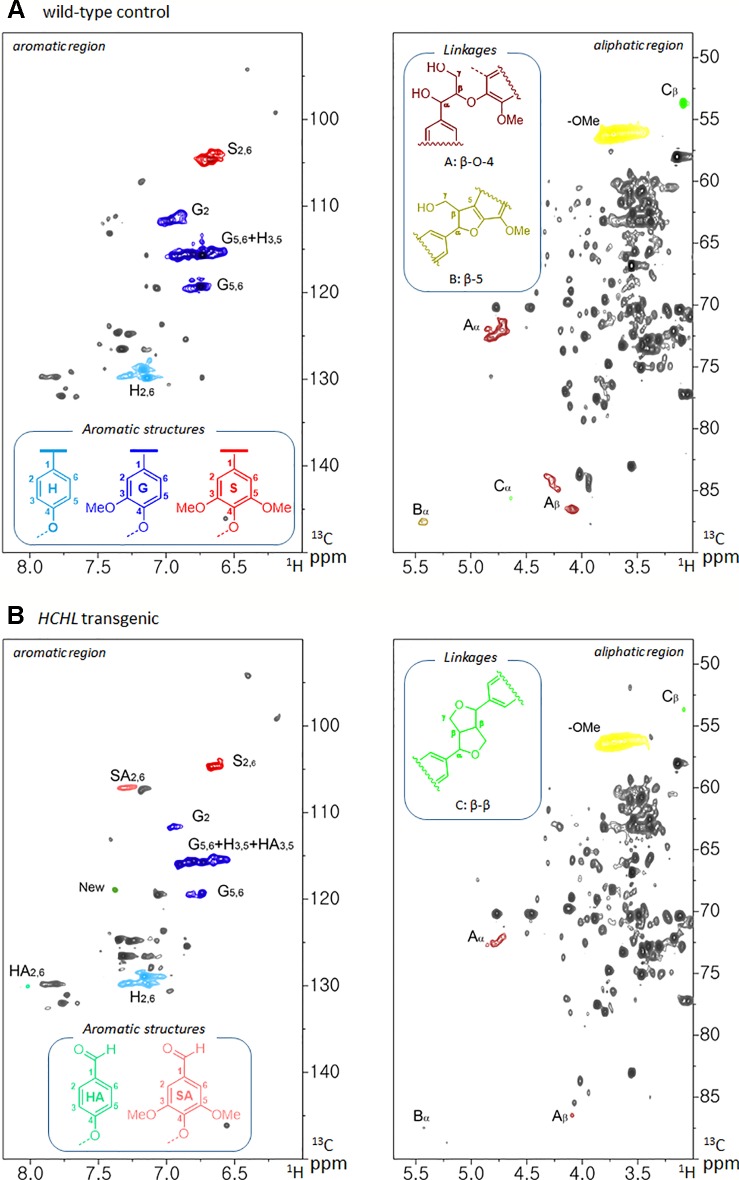
Two-dimensional ^1^H-^13^C heteronuclear single-quantum coherence (2D HSQC) nuclear magnetic resonance (NMR) spectra of isolated lignins from *Arabidopsis* wild-type (WT) **(A)** and hydroxycinnamoyl-CoA hydratase-lyase (HCHL) line **(B)** with main structures of lignin subunits (H, *p*-hydroxyphenyl; G, guaiacyl; S, syringyl; A, β-O-4; B, β-5; and C, β-β).

The isolated lignins were subjected to HSQC NMR analysis to elucidate the structural differences between *Arabidopsis* WT and the HCHL line. The HSQC spectra, respective peak assignments, and lignin substructures of WT and transgenic biomass are shown in **Figure 3**. The HSQC spectra were divided broadly into aromatic (δ_H_/δ_C_ 6.0–8.0/90–150) and aliphatic (δ_H_/δ_C_ 3.0–5.5/50–90) regions. In the aromatic regions of the WT lignin, typical lignin subunits, including *p*-hydroxyphenyl (**H**), guaiacyl (**G**), and syringyl (**S**) were observed. In the lignin isolated from the HCHL line, signal intensities of guaiacyl (**G**) and syringyl (**S**) units were relatively lower. Instead, the aromatic regions of the lignin obtained from the HCHL line had several new correlations compared with WT. For example, the H/C correlations from the oxidized S-unit (**SA**) at 7.3/107.2 ppm and oxidized H-unit (**HA**) at 8.0/130.1 ppm were observed, which is in agreement with the previous study (Eudes et al., 2012). It is also noted that the relative S/G levels of both lignins were similar (0.17 for the WT and 0.19 for the HCHL line), although the engineered lignin exhibited some structural changes.

The aliphatic sidechain region provides information about the structural types and distribution of interunit linkages of the lignin fraction (Kim and Ralph, 2010). HSQC spectra of the WT and engineered lignin showed correlations corresponding to β-ether (β-O-4, substructure **A**), phenylcoumaran (β-5, substructure **B**), and resinol (β-β, substructure **C**) units. As shown in the figure, both lignins isolated from WT and HCHL line exhibited the typical lignin sidechain hydrogen and carbon resonances, although the signal intensities of the engineered lignin were marginally lower than that from WT lignin.

The molecular weight distribution of isolated lignins was measured, and the results are presented in **Table 2**. As seen, the molecular weight of lignins from WT and HCHL line are marginally different (2,853 Da for WT and 2,973 Da for HCHL). Together with the data from biomass compositional analysis, this result suggests that the expression of the *HCHL* gene did not significantly affect lignin content, although some non-traditional side-chain-truncated units were observed.

**Table 2:** Molecular weight distribution of isolated lignins from wild-type (WT) and hydroxycinnamoyl-CoA hydratase-lyase (HCHL) transgenic lines.

	WT	HCHL transgenic
Mw	2853	2973
PDI	3.1	3.3

### Biomass Pretreatment Using Bio-Derived Deep Eutectic Solvent and Enzymatic Saccharification

For biomass pretreatment, the DES (ChCl-VAN) was synthesized at a 1:2 molar ratio. As previously reported, the eutectic point of ChCl-VAN was found to be 55–60°C, which is significantly lower than that of ChCl (302°C) and VAN (81–83°C) (Kim et al., 2019). The pretreatment of WT and transgenic *Arabidopsis* with ChCl-VAN was conducted at 80°C for 3 h. It is noted that the pretreatment conditions used in this work are relatively milder compared with other conventional pretreatment methods (e.g., dilute acid- or organosolv pretreatment) that are typically conducted between 100 and 250°C with catalyst (Wyman et al., 2005; Agbor et al., 2011; Wyman et al., 2013). Indeed, it was hypothesized that reduced biomass recalcitrance due to the incorporation of side-chain-truncated monomers in lignin of engineered plants would facilitate the release of sugars under low-severity pretreatment conditions. After DES pretreatment, 58.0% (WT) and 49.0% (HCHL) of the biomass was recovered. Compositional analysis of the pretreated biomass was conducted and the amount of glucan, xylan, and lignin was as follows; 40.7% (WT) and 40.6% (HCHL) for glucan, 13.2% (WT) and 13.3% (HCHL) for xylan, and 25.0% (WT) and 18.2% (HCHL) for lignin. It also revealed that lignin removal was found to be 33.6%, and 54.7% from WT and HCHL line, respectively. The pretreated solid was enzymatically hydrolyzed for 72 h at 50°C.

**Figure 4:**
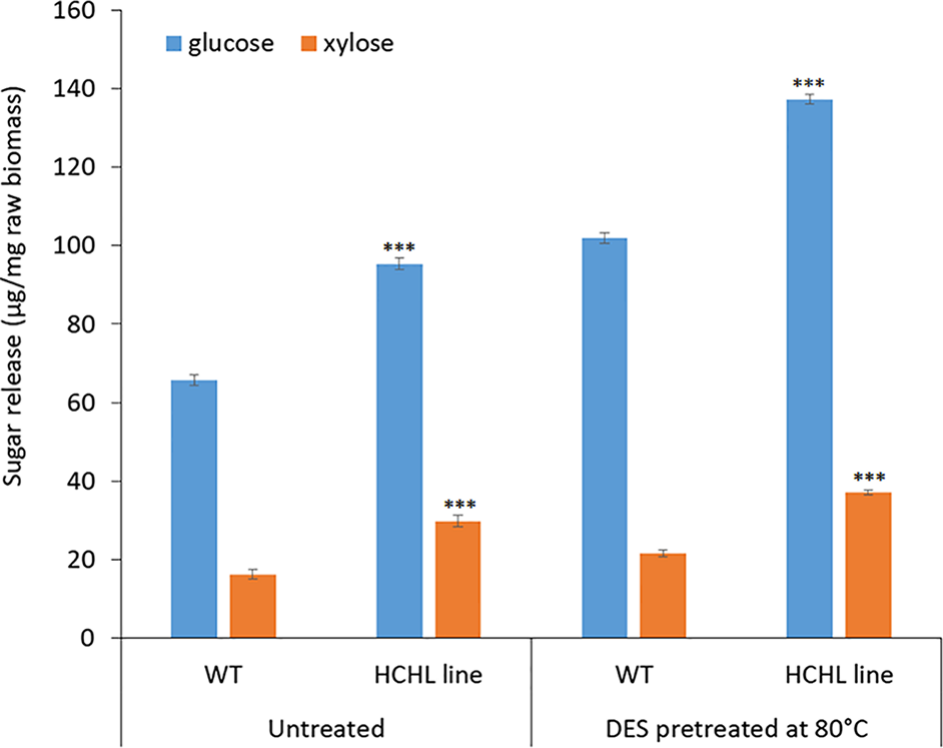
Sugar yield (mean ± standard deviation of three independent tests) from the wild type (WT) and hydroxycinnamoyl-CoA hydratase-lyase (HCHL) lines before and after bio-derived deep eutectic solvent (DES) pretreatment at 80°C (statistical significance from WT ***P < 0.001).

**Figure 4** illustrates the sugar yields after ChCl-VAN pretreatment of biomass from the WT and HCHL lines followed by enzymatic saccharification. Glucose release for WT was 102 μg/mg untreated biomass after ChCl-VAN pretreatment, whereas the amount of glucose released from the engineered biomass was significantly higher (+ 34.3%) and reached 137 μg/mg untreated biomass. This clearly indicates that a strategic expression of the HCHL gene in biomass reduced lignin-associated recalcitrance and resulted in enhanced cell wall digestibility. Similarly, xylose release from the HCHL line was 37 μg/mg untreated biomass, which is 68% higher than that from the WT. The enhanced saccharification was also reported from HCHL-engineered *Arabidopsis* (+78% reducing sugars after hot water pretreatment at 120°C, +31% reducing sugars after 1.2% H_2_SO_4_ pretreatment at 120°C, and +71% reducing sugars after 0.25% NaOH pretreatment at 120°C) (Eudes et al., 2012). Although more comprehensive research is required to optimize DES-assisted pretreatment to maximize the sugar release, this work demonstrates that pretreatment using lignin-derived DES is effective on lignocellulosic biomass.

### Cell Wall Ultrastructural Morphology

**Figure 5:**
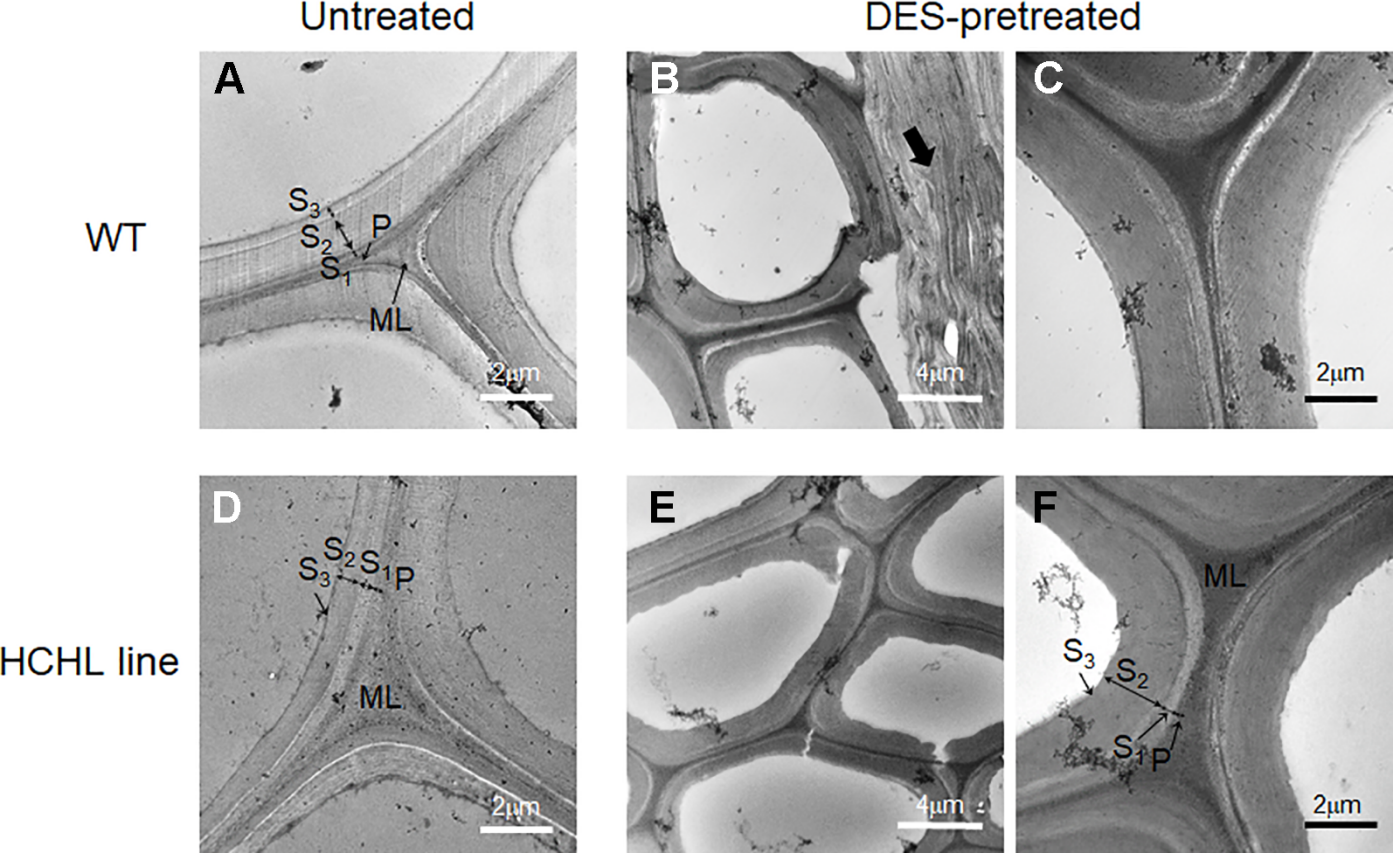
TEM micrographs of cross-sections of wild type (WT) **(A**–**C)** and hydroxycinnamoyl-CoA hydratase-lyase (HCHL) lines **(D**–**F)** before **(A** ,**D)** and after ChCl-VAN pretreatment at 80°C **(B**, **C**, **E**, and **F)**.

Electron microscopic analysis is widely used to obtain high-resolution information on the lignin distribution in biomass cell walls, which can be visualized by staining with potassium permanganate (KMnO_4_) (Koch and Schmitt, 2013). **Figure 5** shows the TEM micrographs of intact stems from WT (**Figure 5A**) and HCHL lines (**Figure 5D**) before and after DES pretreatment at 80°C, in which lignin is selectively stained with 1% KMnO_4_ and darker areas indicate higher lignin concentration.

Regarding the untreated samples, the different layers of fiber and vessel cell walls from both WT and HCHL lines exhibit general staining, including the middle lamella (ML), primary wall (P), and secondary wall (S_1_, S_2_, and S_3_ layers). Particularly, the middle lamella portion is highly reactive to the staining which indicates higher lignin concentration. Previously, it was reported that the HCHL transgenic has a cell wall structure similar to that of WT using light microscopy and stainings such as toluidine blue, Maule, and phloroglucinol-HCl (Eudes et al., 2012). After the DES pretreatment, TEM images of cell wall structures showed little difference for both stem samples. The middle lamella, primary wall, and secondary wall layers are clearly observed for both genotypes, and the middle lamella portion contains higher lignin content than the secondary wall. Considering that the middle lamella lignin is relatively tougher to be removed compared to that in the primary and secondary cell walls (Terashima and Fukushima, 1988; Doherty et al., 2010), the lignin removal after DES pretreatment likely occurs in the primary and secondary cell walls.

Interestingly, some parts of thin wall cells such as in parenchyma cells were compressed and delamination zones were observed for both samples after DES-assisted pretreatment (arrow in **Figure 5B**). This is quite surprising because common DESs have negligible vapor pressure, and the pretreatment conditions used in this work are relatively mild. It is speculated that the cell wall delamination during the pretreatment is likely due to lignin dissolution in the DES, resulting in a loosened fiber cell wall with delamination. Changes in cell wall architecture by DES pretreatment, such as delamination, and possibly an increase in the porosity are main factors for enhanced digestibility (Donohoe et al., 2011). Taken together, the TEM images did not show any distinct differences between unpretreated WT and HCHL lines at the cell wall ultrastructure scale, implying that the enhanced efficiency of pretreatment and digestibility in the case of HCHL-engineered plants should be discussed at the molecular scale.

### Computational Study

**Figure 6:**
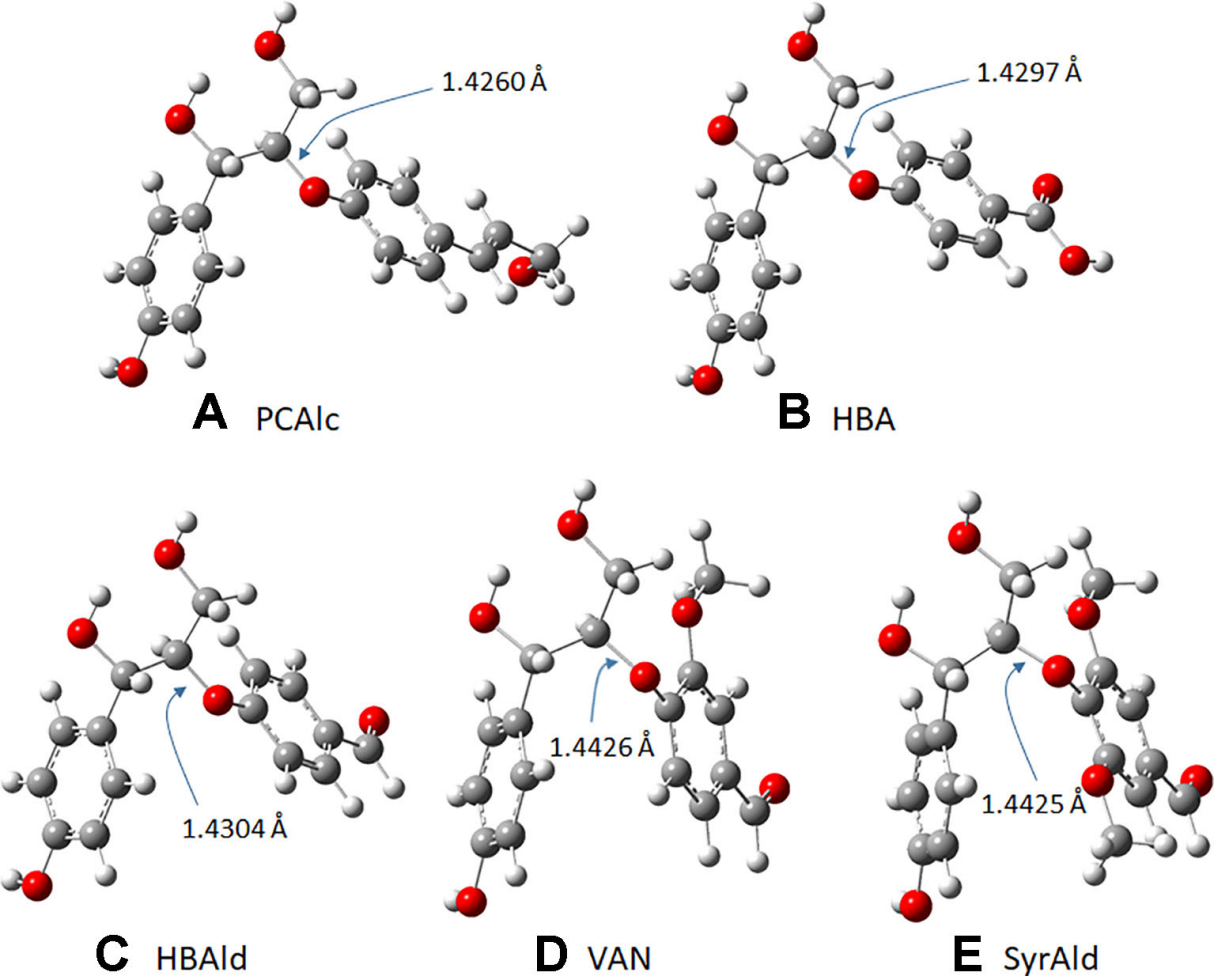
**(A–E)** Optimized geometry of five lignin model dimers and dissociating bond (C_β_-O) length.

As observed above, the strategic introduction of the HCHL gene into lignifying tissue reduced biomass recalcitrance *via* incorporation of side-chain-truncated C_6_C_1_ lignin monomers, which led to enhanced saccharification yields. It was hypothesized that incorporation of C_6_C_1_ monomers makes lignin structure chemically more amenable. To validate this hypothesis, we employed the DFT-based computational study to compute a kinetic quantity of different lignin structures (Socha et al., 2014). Model dimers with aryl-ether linkage found in the typical WT lignin structure and engineered line were used to understand chemical reactivity. Normal β-O-4 structure containing *p*-coumaryl alcohol end group and those with side-chain-truncated monomers (C_6_C_1_: HBA, HBAld, and SyrAld) were computed to find the optimized geometry. **Figure 6** depicts the optimized structures and the C_β_-O bond length in the four lignin model dimers. In this work, the C_β_-O bond length was particularly investigated because it is the most frequent linkage found in the lignin macromolecule and also the weakest linkage that is easily cleaved under heated reaction (Kim et al., 2011). As shown in **Figure 6**, the DFT calculations show that the bond length of C_β_-O in PCAlc dimer is 1.4260 Å. In the case of model dimers with a shorter C_1_ side chain, the dissociating bond length noticeably elongated. For example, the C_β_-O bond length increased to 1.4297, 1.4340, 1.4426, and 1.4425 Å with HBA, HBAld, VAN, and SyrAld, respectively. This result indicates that it requires a lower bond dissociation energy for the ether linkage when the C_6_C_1_ monomers are incorporated into lignin structure.

**Figure 7:**
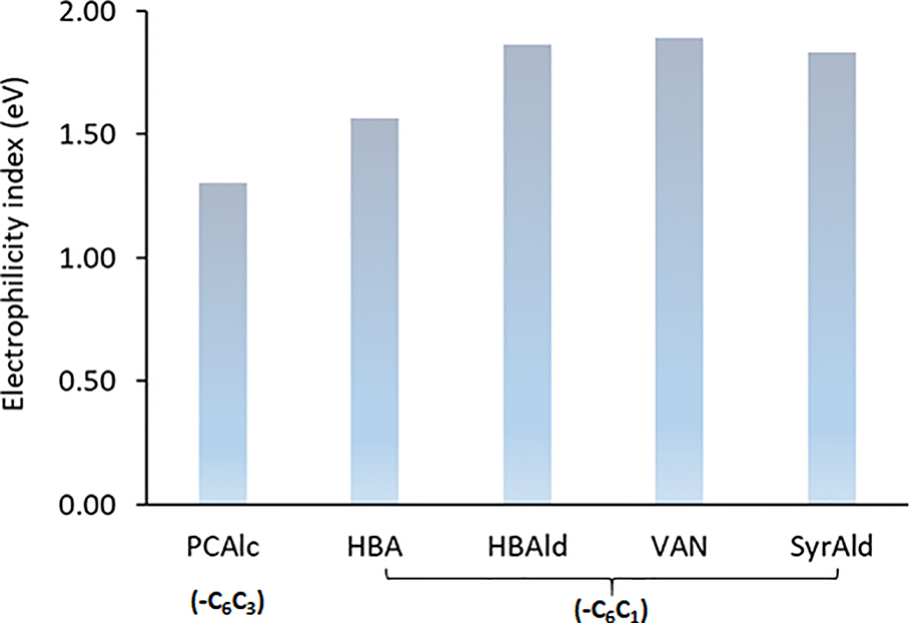
Electrophilicity index of dimeric model compounds.

The electrophilicity index was also calculated to assess the chemical reactivity of dimeric model compounds, and the results are given in **Figure 7**. The electrophilicity index of common lignin dimeric structure with *p*-coumaryl alcohol end group (PCAlc) was calculated to be 1.30 eV. Interestingly, the values of four dimeric model compounds with a shortened C_1_ side-chain monomer end group were considerably higher than that of a conventional lignin structure, representing 1.56 (+20.2%), 1.86 (+43.1%), 1.89 (+45.3%), and 1.83 (+40.8%) with HBA, HBAld, VAN, and SyrAld, respectively. It is evident that lignin dimers with C_6_C_1_ monomers have a higher electrophilicity index, suggesting that these structures are chemically more reactive than the typical β-O-4 structure (Shi et al., 2016). The results from DFT computational study support the hypothesis that lignin structure in the HCHL line is more susceptible to biomass pretreatment.

## Discussion

Throughout the last decades, cell wall engineering has been a critical approach to reduce biomass recalcitrance by altering lignin structures. Perturbations of genes on the biosynthesis pathway of lignin result in significant structural changes, which allows designing readily tractable biomass structures for the production of biofuels and chemicals (Ralph et al., 2019). The strategic expression of HCHL in lignifying tissues of *Arabidopsis* resulted in the overproduction of side-chain-truncated (C_6_C_1_) lignin monomers incorporated in lignin as end-groups (Eudes et al., 2012). 2D HSQC NMR analysis confirms that isolated lignin from HCHL transgenic biomass contains a significant amount of oxidized C_6_C_1_ units. Considering the similar lignin content with a slight difference in molecular weight of isolated lignin, as well as comparable ultrastructural morphologies between the WT and HCHL lines, we propose that engineering the biosynthesis of C_6_C_1_ monomers in-planta is an effective approach to modify lignin without any agronomic penalty.

In this work, VAN, an oxidized C_6_C_1_ unit found in HCHL plants, was used to synthesize ChCl-VAN, a renewable DES that was previously shown to be effective for biomass pretreatment (Kim et al., 2018). Furthermore, integration of lignin-derived DES with HCHL-engineered biomass offers opportunities to move closer towards achieving a closed-loop biorefinery (Kim et al., 2019). The pretreatment of WT and HCHL biomass using ChCl-VAN showed that lignin removal from transgenic biomass was higher, resulting in enhanced saccharification efficiencies. Considering that lignin content in both samples is quite similar, the improvement of biomass digestibility for the HCHL line is associated with 1) significant structural alteration in lignin, and 2) the higher reactivity of short-side chain lignin monomers.

Regarding the chemical properties of lignin derived from HCHL plants, DFT-based calculation revealed higher reactivity for structures corresponding to C_6_C_1_ monomers linked to lignin. Moreover, lignin with side-chain-truncated monomers requires less energy for the cleavage of β-aryl ether bond, which is typically necessary for lignin fractionation during biomass pretreatment. Previously, lignins rich in H-units were computationally found to be more reactive than other types of lignins (G- and S-unit) (Shi et al., 2016), and transgenic biomass consisting of H-lignin yielded higher sugars upon saccharification (Bonawitz et al., 2014). As discussed above, the more reactive nature of C_6_C_1_ monomers with aldehyde or carboxylic acid functionalities that form lignin end-groups contributed to the increased pretreatment efficiency followed by higher saccharification yield.

It is noted that although both empirical and computational results undoubtedly support the hypothesis, more work is necessary to gain a far-reaching insight of structural modification resulting from the expression of HCHL. However, the pretreatment of transgenic biomass using a bio-derivable DES offers opportunities to operate reactors with reduced amount of energy and chemicals, which is highly desirable in developing a sustainable bioeconomy.

## Conclusions

Plant cell wall engineering has been developed for the production of biofuels and renewable chemicals. In this study, we strategically expressed the HCHL gene in *Arabidopsis* to reduce the degree of lignin polymerization *via* incorporation of side-chain-truncated monomers in lignin polymer ends. The transgenic *Arabidopsis* yielded higher levels of fermentable sugars compared to WT plants when pretreated with the lignin-derived DES at mild conditions. The results of this work clearly indicate that interfering with the lignin biosynthetic pathway has the potential to improve the conversion of biomass into biofuels and other intermediate products. Together with the development of tailor-made biomass that is more amenable to chemical processes, biomass pretreatment using a renewable DES could make the biofuel industry more economically feasible in the future.

## Data Availability Statement

The datasets analyzed in this article are not publicly available. Requests to access the datasets should be directed to KHK, kwanghokim@kist.re.kr.

## Author Contributions

KHK conceived and designed the experiments. KHK, YW, MT, and AE performed the experiments. KHK, CGY, CSK, and JS analyzed the data of the experiments. KHK, MT, AE, and CGY drafted manuscript. All authors read and approved the final manuscript.

## Funding

This work is supported by the Korea Institute of Science and Technology–The University of British Columbia Biorefinery on-site laboratory project. NMR analysis is supported by SUNY ESF and NIH Shared Instrumentation Grant 1S10OD012254. This work was part of the DOE Joint BioEnergy Institute (http://www.jbei.org) supported by the U. S. Department of Energy, Office of Science, Office of Biological and Environmental Research, through contract DE-AC02-05CH11231 between Lawrence Berkeley National Laboratory and the U.S. Department of Energy.

## Conflict of Interest

The authors declare that the research was conducted in the absence of any commercial or financial relationships that could be construed as a potential conflict of interest.
